# Successful Replantation Despite Improper Storage of Amputated Thumb: A Case Report

**DOI:** 10.5704/MOJ.2203.019

**Published:** 2022-03

**Authors:** J Gunasagaran, YY Tan, TS Ahmad

**Affiliations:** NOCERAL, Department of Orthopaedic Surgery, Universiti Malaya, Kuala Lumpur, Malaysia

**Keywords:** digit replantation, improper storage, contact on ice, immersion in water, case report

## Abstract

Replantation of fingers is highly complex and technically challenging. Surgeons are serious with their selection criteria as many factors are involved in determining good surgical outcome. Improper storages of amputated parts are usually denied the option for replantation. We report a 42-year-old lady who was assaulted with a machete and presented with total amputation of left thumb. The amputated thumb was stored in a plastic bag directly on ice cubes which eventually melted; thumb immersed in water for two hours. On examination, the amputated thumb was neither macerated nor frozen. Replantation was attempted and was successful. There are limited reports on proper methods of storage of amputated fingers pertaining to daily practical scenario. Yet, it is a strict criterion for surgeons in attempting replantation. Direct contact of amputated fingers on ice and immersion in hypotonic solutions leads to irreversible tissue damage. In our case, two hours of unfavourable storage did not affect surgical outcome. In conclusion, clinical assessment of the amputated part is essential in deciding for replantation. Combination of direct contact with ice and immersion in hypotonic solution for two hours should not be a contraindication for replantation.

## Introduction

Replantation of fingers is highly complex, delicate, and technically challenging. Surgeons are serious with their selection criteria as many factors are involved in determining good surgical outcome. Success of replantation depends on the level and nature of injury itself, age and vascular condition of patients, quality of micro anastomosis and postoperative care^1-3^.

Storage of the amputated digit under low temperature is an important factor in determining good surgical outcome^1^. Thus, surgeons frequently hesitate to attempt replantation if the storage is not physiological. There are two basic methods proposed on storage of amputated fingers; either wrap it with moistened cloth with normal saline or Ringer’s lactate solution, place it in a plastic bag and keep it on ice; or immerse it into normal saline or Ringer’s lactate solution in a plastic bag and keep it on ice^4^. We report a case of successful replantation despite unfavourable storage conditions.

## Case Report

**Fig. 1:**
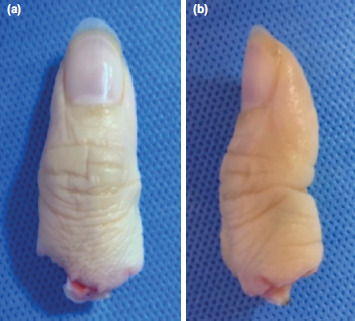
(a) and (b) Anteroposterior and lateral view of amputated left thumb, at the level base of proximal phalanx.

A 42-year-old lady was assaulted with a machete and presented with total amputation of left thumb at base of proximal phalanx. The amputated thumb was found on the road. A passerby helped to wash it under tap water and placed it in a plastic bag with ice cubes. She presented at Emergency Unit about two hours later where the ice cubes had melted, and the thumb was completely immersed in ice water. The thumb was immediately rewashed with normal saline. The edges appeared sharply cut with minimal soft tissue damage. There was neither maceration nor frostbite (Fig. 1). The amputated thumb was wrapped with moist gauze and stored in ice box. We hesitated to replant due to improper storage. After careful patient counselling, we decided to proceed with surgery with patient’s consent.

**Fig. 2:**
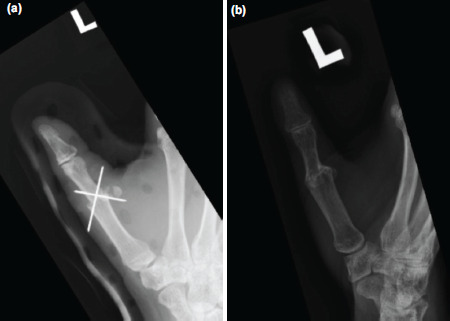
Radiographs of left thumb anteroposterior view (a) day 2 and (b) 1 year post-surgery.

After general anaesthesia, left upper limb was prepped and tourniquet was inflated. Two teams of surgeons debrided and explored both the stump and the amputated part simultaneously; fusion of metacarpophalangeal joint performed with K-wires (Fig. 2). Flexor pollicis longus and digital arteries were repaired. Torniquet was released within two hours and veins were allowed to ooze. A stat dose heparin 5000 IU was given intravenously. Subsequently, extensor pollicis longus, three dorsal veins and both digital nerves were repaired. All micro-anastomosis and nerve coaptations were done with Nylon 10-0 under microscope.

**Fig. 3:**
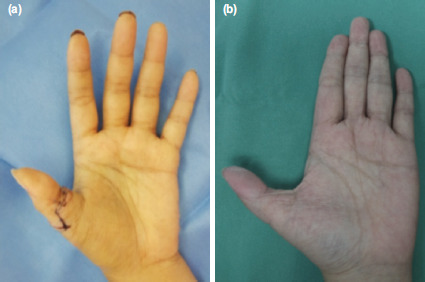
Post-replantation at (a) two weeks and (b) one year follow-up.

Post-operatively, patient was well-hydrated. Heparin infusion and aspirin were started and the thumb was kept warm under a heat lamp. Heparin was discontinued after five days and allowed discharge with aspirin for two weeks. Antibiotics were completed for a week. Wound healed well at two weeks post-surgery (Fig. 3) and passive range of motion exercises were started. Radiographs showed union after two months; thus, K-wires were removed and active range of motion exercises commenced. At one year follow-up, she could oppose her fingers despite shortening of 1cm. Active range of motion at interphalangeal joint was 5° to 45°. Grip strength was 22kg and pinch strength was 4kg; almost similar to the opposite hand. Sensation at fingertip on radial aspect was normal, ulna aspect was reduced with intact protective sensation (3.61/0.4g Semmes-Weinstein monofilament). She expressed her utmost gratitude for the treatment received.

## Discussion

Many literatures have emphasised the importance of ischaemic time in determining the success of replantation of extremities. Storage of amputated digits at room temperature for two hours (warm ischaemic time) is accepted as safe^4^. Hayhurst *et al* reported a case of successful replantation of a digit stored 3 hours at 23°C^5^. Replantation should not be attempted if warm ischaemic time exceeds 6-8 hours, especially if muscle tissues are present. Storage at 4°C (cold ischaemic time) is considered the best and replantation can be performed even after 24 hours^4,5^. This is possible as the temperature halts tissue metabolism and autolysis; thus, reduces metabolic acidosis and reduces bacterial growth^4^.

However, storage at 0°C causes freezing leading to irreversible tissue damage. Even with a successful micro anastomosis, good perfusion can be achieved but eventually, the extremity cannot be salvaged. VanGiesen *et al* reported failures of all his replantations in this storage condition^4^. Thus, direct contact of amputated digit on ice is prohibited. Total immersion of the amputated digit in Ringer’s lactate or normal saline at 4°C is a better method of storage as uniform cooling ensured. However, the solution must be isotonic. Immersion in hypotonic solutions for example tap water may predispose to excessive tissue oedema and maceration secondary to cell lysis and osmosis.

There are limited reports on proper method of storage of amputated fingers pertaining to daily practical scenario. Yet, it is a strict criterion for surgeons in attempting replantation; fearing of failure and unwanted complications. Both the storage methods recommended by VanGiesen *et al*^4^, needs isotonic solution either to be soaked with cloth and wrapped or immersed totally.

Mimicking daily practical setting, Ringer’s lactate and normal saline are not readily available in public. In addition, the temperature of the stored digit cannot be controlled or fixed to the ideal 4°C. Thus, surgeons should anticipate that the amputated digit would have not been stored in an ideal method. The surgeons should not deem it unsuitable for replantation and deny the opportunity by assessing this criterion alone. Two hours of improper storage of amputated digit can still be salvaged provided the other factors are favourable. Therefore, clinical assessment of the amputated digit is crucial. If the digit appears neither frozen nor macerated, replantation can be attempted. Our replantation was successful despite the unfavourable condition and patient achieved satisfaction functionally and aesthetically.

In conclusion, clinical assessment of the amputated part is essential in deciding for replantation. Combination of direct contact with ice and immersion in hypotonic solution for two hours should not be a contraindication for replantation.
